# Suicide deaths in Ireland before and during the first year of COVID-19: What changed?

**DOI:** 10.1016/j.xjmad.2026.100210

**Published:** 2026-10-16

**Authors:** Leigh Huggard, M. Isabela Troya, Gemma Cox, Katerina Kavalidou, Paul Corcoran, Elaine McMahon

**Affiliations:** a School of Public Health, University College Cork, Cork, Ireland; b National Suicide Research Foundation, Cork, Ireland; c National Office for Suicide Prevention, Dublin, Ireland

**Keywords:** Suicide, Determinants, COVID-19, Mental health, Physical health

## Abstract

Public health emergencies such as the COVID-19 pandemic have raised concerns regarding their impact on suicide risk, through increased social isolation, healthcare disruption, and economic stress. International evidence on suicide trends during the first year of the pandemic is mixed; there is limited peer-reviewed research examining suicide deaths in Ireland during this period. The study aimed to compare sociodemographic, mental health, and physical health characteristics of individuals who died by suicide in Ireland before and after the onset of the COVID-19 pandemic, and to identify the distinguishing characteristics of individuals whose deaths involved documented COVID-19-related risk factors. Data were drawn from the Irish Probable Suicide Deaths Study (IPSDS), comprising coronial records of all probable suicides in Irelands from 2015 to the end of 2020 (N = 3620). Descriptive statistics and chi-square tests compared characteristics of suicide deaths before (n = 3156) versus after (n = 464) the COVID-19 lockdown. A binary logistic regression compared the differences in characteristics of individuals with documented COVID-19-related risk factors to those without such factors. Compared with the pre-pandemic cohort, individuals who died by suicide during the lockdown period in 2020 were more likely to be female, have a documented physical health concern, and were more likely to exhibit behavioural changes prior to death. Individuals with mental and physical health concerns were more likely to have COVID noted as a significant factor in their deaths. The COVID-19 pandemic was associated with modest but important shifts in the characteristics of suicide deaths in Ireland, particularly among individuals with physical health conditions.

## Highlights

• Suicide deaths during the first COVID lockdown were more likely to involve females and those with physical health concerns.
• Study and work-related stressors were less prominent following lockdown.
• Mental and physical health concerns were more common in COVID-related suicide deaths.

## 1 Introduction

Public health emergencies can impact population mental health and suicide risk and accentuate key risk factors such as social isolation, reduced access to mental health services, financial insecurity, and heightened fear and uncertainty [1], [2]. Learnings from previous public health emergencies, such as the COVID-19 pandemic, can be invaluable in informing planned responses to potential future public health emergencies. While the specific drivers of suicide may change depending on the nature of the crisis, the patterns of how risk shifts across demographic groups offer critical insights into the resilience of social safety nets and mental health systems. Understanding these shifts is essential not only for pandemic preparedness but for developing adaptive suicide prevention strategies that can be deployed during any period of acute societal instability.

COVID-19 was declared a public health emergency of international concern by the World Health Organisation in January 2020 and designated a pandemic in March 2020 [3]. In response, countries adopted varying approaches to contain the virus [4]. Concerns about a potential increase in suicide deaths during the COVID‑19 pandemic were raised early, given the widespread presence and amplification of known suicide risk factors [5], [6]. In addition to reduced social support and enforced social isolation, the pandemic was associated with significant economic disruption, job loss, housing precarity, and increased exposure to psychosocial stressors [6], [7]. Disruptions to routine healthcare and community‑based services further heightened concerns about reduced access to mental health assessment and ongoing care, particularly for individuals with pre‑existing mental health conditions [5].

International evidence on suicide trends during the first year of the COVID‑19 pandemic has been mixed. Several countries reported no increase, or even temporary decreases, in suicide deaths during 2020 [8], [9]. Conversely, emerging evidence suggests increases among specific population groups, such as children and adolescents [10], and older adults [11]. These divergent findings highlight the importance of examining differential impacts across sociodemographic groups.

Ireland provides a relevant context for investigating suicide during the COVID‑19 pandemic. Ireland implemented some of the longest and most restrictive public health measures in Europe during 2020 [12]. While these measures contributed to Ireland recording one of the lowest excess death rates [13], they also entailed prolonged restrictions on social contact, mobility, education, and employment. Simultaneously, the Irish government introduced a range of economic and social protection measures aimed at mitigating financial stressors. Understanding how these concurrent risk and protective factors influenced suicide mortality is essential for informing future public health emergency planning. While Irish data show suicide rates remained relatively unchanged before and during the first year of the pandemic, [14], limited research examines whether this varied by characteristic, and key risk factors that shaped outcomes.

### 1.1 Aims

• To compare the sociodemographic, mental health, and physical health characteristics of individuals who died by suicide before and after the onset of the COVID-19 pandemic (2015–2020)
• To describe the characteristics of individuals whose coronial files included documented COVID-19-related risk factors for suicide, and to compare these individuals to those without documented COVID-19-related risk factors.

## 2 Methods

### 2.1 Data

The Irish Probable Suicide Deaths Study (IPSDS) records detailed characteristics of all individuals who died by suicide in Ireland between 01/01/2015 and 31/12/2020 (*N* = 3630) [15]. Suicide deaths were defined where there was either a verdict of suicide or where the death was in line with the Rosenberg [16] criteria of suicide. Data were collected across all coroners' districts nationally. Coronial files were examined by nurse researchers, who extracted relevant information compiled as part of the death-investigation process.

### 2.2 Sample and data cleaning

The IPSDS contains sociodemographic variables (e.g., age, sex, living situation, employment status), health-related indicators (e.g., mental health, physical health, substance misuse, prior self-harm), and documented risk factors (e.g., behavioural changes, study/work stress, COVID-19-related factors). Risk factors were researcher determined based on information supplied during the inquest. For example, behavioural changes were defined as any reports of significant changes in an individual’s behaviour during the period leading up to their death. As coroners did not follow a structured data extraction methodology, there was a range of missingness, (0% for sex, age, and method of suicide; 3.0% for relationship status; 9.3% for living situation; 15.7% for employment status; 43.8% for socioeconomic status). Risk-factor variables, such as prior self-harm, drug and/or alcohol misuse, study/work stress, and COVID-19-related factors, were coded as binary (1 = documented presence, 0 =no documented presence). Low frequency categories were collapsed to facilitate statistical comparison. Age was grouped categorically.

The study included all individuals who died by suicide in Ireland before (*n* = 3156) and after (*n* = 464) the onset of the COVID-19 pandemic, defined as the 12th March 2020, for the study period of 2015–2020. This date corresponds to the official announcement by the Irish Taoiseach (Prime Minister) introducing national public health measures and formally acknowledging the pandemic.

### 2.3 Analysis methods

Descriptive statistics summarised the sociodemographic, mental, and physical health characteristics of study individuals who died by suicide before versus after the onset of the pandemic. Group comparisons were conducted using chi-square tests.

Descriptive statistics compared individuals with versus without documented COVID-19-related risk factors. Initial chi-square comparisons, frequencies, and percentages were calculated. Open-text descriptions of COVID-19-related risk factors were grouped into thematic categories and are described below.

A binary logistic regression assessed differences between those with and without COVID-19-related risk factors, with sex, age category, mental health status, and physical health status included as independent variables, and COVID risk factor status as the dependent variable. These variables were selected due to both theoretical relevance and significance in the initial chi-square exploratory analyses. Adjusted odds ratios with 95% confidence intervals were reported.

Analyses were performed in RStudio v4.4.0.

## 3 Results

### 3.1 Characteristics of the suicide cohort before and after the onset of the COVID-19 pandemic (2015–2020)

Descriptive findings (Table 1) indicated that, compared to the cohort who died by suicide before the beginning of the COVID-19 lockdown period, individuals who died by suicide in post-lockdown 2020 were more likely to be female (*p* = .004). However, there were no significant differences in age group or other sociodemographic characteristics between the cohorts.

**Table 1:** Characteristics of the national suicide cohort before and during the COVID-19 lockdown period (2015–2020).

		**Pre-COVID-19 lockdown (2015 – March 2020)**		**Post-COVID-19 lockdown (March – December 2020)**					
		***n***	***%***	***n***	***%***	$\chi^{2}$	***df***	***p***	**Effect size (Cramer’s V)**
**Gender**	Female	2376	75%	320	69%	8.17	1	.004**	0.05
	Male	780	25%	144	31%				
**Age category**	10–24	400	13%	59	13%	0.27	3	.965	0.01
	25–44	1235	39%	178	38%				
	45–64	1171	37%	172	37%				
	65 +	350	11%	55	12%				
**Had a physical health condition**	Yes	578	18%	118	25%	12.74	1	< .001***	0.06
	No	2578	82%	346	75%				
**Had a mental health condition**	Yes	2092	66%	287	62%	3.33	1	.068	0.03
	No	1064	34%	177	38%				
**Prescription for mental health medication**	Yes	1212	38%	161	35%	2.20	1	.138	0.03
	No	2415	77%	350	75%				
**Prior self-harm**	Yes	741	23%	114	25%	0.21	1	.647	0.01
	No	2415	77%	350	75%				
**Method of suicide**	Drowning	367	12%	29	6%	14.64	3	.002**	0.06
	Hanging	1978	63%	324	70%				
	Other	420	13%	60	13%				
	Poisoning	391	12%	51	11%				
**Drug/alcohol**	Yes	838	27%	123	27%	0.00	1	.999	0.00
	No	2318	73%	341	73%				
**Employment status**	Employed	1088	34%	177	38%	4.70	3	.195	0.04
	Student	232	7%	37	8%				
	Unemployed	786	25%	100	22%				
	Retired/unable to work/Not working	558	18%	72	16%				
**Socioeconomic status**	Higher	306	10%	47	10%	2.78	2	.250	0.04
	Middle	751	24%	123	27%				
	Lower	716	23%	92	20%				
**Relationship status**	Co-habiting	183	6%	15	3%	6.05	3	.109	0.04
	Married/Civil partnership	943	30%	135	29%				
	Separated/divorced/widowed	346	11%	48	10%				
	Single	1592	50%	250	54%				
**Living situation**	Alone	774	25%	112	24%	0.09	3	.993	0.01
	Family	913	29%	136	29%				
	Other shared (e.g., with friends)	120	4%	17	4%				
	Partner/children/both	923	29%	138	30%				
**Have children**	Yes	528	17%	80	17%	0.02	1	.894	0.00
	No	1994	63%	294	63%				
**Changes in behaviour**	Yes	468	15%	192	41%	189.50	1	< .001***	0.23
	No	2688	85%	272	59%				
**Study/work stress**	Yes	275	9%	26	6%	4.73	1	.030*	0.04
	No	2881	91%	438	94%				

Note: ***p < .001, **p < .01, *p < .05; Cramer’s V 0.10 = small, 0.30 = medium, 0.50 = large.

The post-lockdown 2020 cohort were significantly more likely to have a documented physical health concern (*p* < .001), but not more likely to have a mental health concern or a documented history of self-harm or drug/alcohol dependency.

There was a significant difference in suicide method following the onset of the pandemic (*p* = .002), with a lower proportion of deaths by drowning in the post-lockdown period. Individuals in the post-pandemic cohort were significantly more likely to have documented changes in behaviour prior to death (*p* < .001), and significantly less likely to have documented education-related or work-related stressors (*p* = .030).

### 3.2 Characteristics of individuals with documented COVID-19-related risk factors

Of the 464 individuals who died by suicide in post-lockdown 2020, 64 (13.8%) had COVID-19-related factors noted as a probable contributor to their death. The most frequent factor was COVID-19 restrictions and lockdown (67%, n = 43; e.g., “*Effect of isolation due to Covid 19 restrictions on mental health*”), followed by general/non-specific COVID-19-related factors (16%; *n* = 10), financial/employment impacts (9%; e.g., “*loss of business due to Covid restrictions*”), healthcare-related factors (6%; e.g., “*Unable to get a GP appointment due to Covid*”), and high-risk or positive COVID-status (6%; e.g., “*High risk for COVID*”).

Descriptive findings (Table 2) indicated that individuals with COVID-19-related risk factors were significantly more likely to be aged over 65 years, female, have documented physical and mental health concerns, have received a prescription for mental health medication, and have documented behavioural changes prior to their deaths.

**Table 2:** Cohort of individuals who exhibited COVID-related determinants of suicide vs those without, 2015–2020.

		**With COVID-19-related risk factors**		**Without COVID-19-related risk factors**							
		***n***	***%***	***n***	***%***	$\chi^{2}$	***df***		***p***		**Effect size (Cramer’s V)**
**Sex**	Female	25	39%	900	25%	5.57	1		.018*		0.04
	Male	39	61%	2657	75%						
**Age category**	10–24	n < 10	8%	454	13%	13.74	3		.003**		0.06
	25–44	25	39%	1388	39%						
	45–64	18	28%	1325	37%						
	65 +	16	25%	389	11%						
**Physical health condition**	Had a documented physical health concern	24	38%	672	19%	12.87	1		< .001***		0.06
	Did not have a documented physical health concern	40	63%	2887	81%						
**Mental health**	Had a documented mental health concern	54	84%	2325	65%	9.24	1		.002**		0.05
	Did not have a documented mental health concern	10	16%	1231	35%						
**Prescription for mental health medication**	Yes	37	58%	1336	38%	9.44	1		.002**		0.07
	No	27	42%	389	62%						
**Prior self-harm**	Yes	18	28%	837	24%	0.51	1		.477		0.01
	No	46	&2%	2722	76%						
**Method suicide**	Drowning	n < 10	8%	391	11%	1.77	3		.621		0.02
	Hanging	44	69%	2261	64%						
	Other	n < 10	9%	474	13%						
	Poisoning	n < 10	14%	433	12%						
**Drug/alcohol dependency**	Yes	14	22%	462	15%	0.51	1		.477		0.01
	No	50	78%	2609	85%						
**Employment status**	Employed	27	48%	1238	43%	1.50	3		.682		0.02
	Student	n < 10	9%	264	9%						
	Unemployed	13	23%	873	31%						
	Retired/unable to work	11	20%	486	17%						
**Socioeconomic status**	Higher	n < 10	23%	345	17%	5.15	2		.076		0.05
	Middle	14	40%	860	43%						
	Lower	13	37%	795	40%						
**Relationship status**	Co-habiting	n < 10	6%	194	6%	1.83	3		.609		0.02
	Married/Civil partnership	22	35%	1057	31%						
	Separated/divorced/widowed	n < 10	6%	390	11%						
	Single	32	52%	1812	52%						
**Living situation**	Alone	19	31%	867	28%	2.60	3		.458		0.03
	Family	15	25%	1034	34%						
	Other shared (e.g., with friends)	n < 10	7%	133	4%						
	Partner/children/both	23	38%	1039	34%						
**Have children**	Yes	14	25%	595	21%	0.33	1		.565		0.01
	No	42	75%	2248	79%						
**Changes in behaviour**	Yes	36	56%	624	18%	60.68	1		< .001***		0.13
	No	28	44%	2935	82%						
**Study/work stress**	Yes	10	16%	291	8%	3.65	1		.056		0.03
	No	54	84%	3268	92%						

Note: ***p < .001, **p < .01, *p < .05; Cramer’s V 0.10 = small, 0.30 = medium, 0.50 = large.

#### 3.2.1 Logistic regression model

Binary logistic regression indicated that individuals with a mental health concern were 2.6 times more likely to have a documented COVID-19-related risk factor than those without (OR=2.60, 95%CI[1.36, 5.48], *p* = .007). Those with physical-health concerns were 2.2 times more likely to have a documented COVID-19-related risk factor (OR=2.17, 95%CI[1.25, 3.67], *p* = .005).

Sex and age category did not retain significance in the model (female vs. male: *p* = .057; age 65 + vs. 10–24: *p* = .064), suggesting that the observed raw differences in COVID status by age and sex (Table 1) were not retained while controlling for mental and physical health status. Instead, the differences in age and gender may be driven by the higher co-occurrence of mental and physical health conditions within those sociodemographic groups.

## 4 Discussion

This study examined the characteristics of individuals who died by suicide in Ireland before and during the first year of the COVID-19 pandemic. Characteristics remained relatively stable across the pre- and post-pandemic periods, potentially due to buffering effects of social protection measures early in the pandemic. Notably, study and work-related stressors were less prominent following lockdown, potentially reflecting the efficacy of such measures. However, several important changes in characteristics were identified: the post-lockdown cohort were significantly more likely to be female, have a physical health concern, and have documented behaviour changes, suggesting impacts were not uniform but concentrated among specific subgroups. The increased representation of women may reflect gendered experiences of pandemic-related stressors. During the early pandemic, women reported greater COVID anxiety and faced disproportionate socioeconomic pressures, including increased caregiving responsibilities, higher rates of precarious employment, and greater exposure to domestic stressors [17], [18], [19]. Similarly, the increased prevalence of physical health conditions in the post-lockdown cohort may be explained by associated stressors, such as heightened perceived health risk, social isolation, and healthcare disruption [20].

The study also specifically examined a sub-group for whom COVID was a significant factor in their death, who significantly more likely to be female and over 65 years. However, the combined model suggested the COVID sub-group was more likely to have a history of mental and physical illness, whereas there were no sex and age differences. Individuals with pre-existing mental and physical health concerns may have been disproportionally impacted by the pandemic. However, no overall difference in mental health concerns were observed between the pre- and post-pandemic cohorts. It is possible that mental health functioned as a mediating factor for unmeasured factors.

A key strength of this study is the use of complete national population-level coronial data, offering detailed information beyond routine mortality statistics. Limitations include reliance on information recorded during the death investigation process, potentially under-reporting risk factors. The study period was confined to the first year of the pandemic, and suicide patterns may have evolved differently as restrictions changed, vaccination programmes were introduced, and cumulative socioeconomic consequences emerged. Lastly, the study was conducted specifically within an Irish cohort, which may limit the generalisability of the findings to populations outside of Ireland.

The first year of the COVID-19 pandemic in Ireland was associated with modest shifts, rather than broad changes in the characteristics of suicide deaths. Females and individuals with pre-existing physical health conditions were over-represented, while individuals with mental and physical health concerns were more likely to have COVID noted as a significant factor in their deaths.

## CRediT authorship contribution statement

**Leigh Huggard:** Conceptualisation, Formal analysis, Writing – original draft. **Isabela Troya:** Conceptualisation, Writing – original draft. **Gemma Cox:** resources, writing – original draft. **Katerina Kavalidou:** Resources, Writing – review & editing. **Paul Corcoran:** Writing – review & editing. **Elaine McMahon:** Conceptualisation, Writing – original draft.

## Ethics approval

This retrospective database study was conducted in accordance with the ethical standards of the relevant institutional and national research committees and with the 1964 Helsinki Declaration and its later amendments. Ethical approval for the original IPSDS study was obtained from the Ethics Committee of the Irish College of General Practitioners (ICGP) in 2016. Ethical approval for the current study was obtained from the Clinical Research Ethics Committee of the Cork Teaching Hospitals. Data were analysed in de-identified form and all applicable confidentiality and data protection requirements were observed.

## Funding

This work was supported by the Health Research Board, grant number HRB-EIA-2024-011.

## Declaration of Competing Interest

The authors declare that they have no known competing financial interests or personal relationships that could have appeared to influence the work reported in this paper.

## Data Availability

Access to the anonymised and de-identifiable Irish Probable Suicide Deaths Study data may be requested by contacting the HSE National Office for Suicide Prevention (NOSP), email info@nosp.ie. Data requestors will need to sign a data access agreement.
